# Urine metabolomics unravel the effects of short-term dietary interventions on oxidative stress and inflammation: a randomized controlled crossover trial

**DOI:** 10.1038/s41598-024-65742-6

**Published:** 2024-07-03

**Authors:** Digar Singh, Dongwoo Ham, Seong-Ah Kim, Damini Kothari, Yu Jin Park, Hyojee Joung, Choong Hwan Lee

**Affiliations:** 1 Department of Botany and Microbiology, Hemvati Nandan Bahuguna Garhwal University, Srinagar (Garhwal), Uttarakhand 246174 India; 2 Institute of Health and Environment, Seoul National University, Seoul, 08826 Republic of Korea; 3 Division of Strategic Research, The Seoul Institute, Seoul, 06756 Republic of Korea; 4 Department of Biochemistry, Hemvati Nandan Bahuguna Garhwal University, Srinagar (Garhwal), Uttarakhand 246174 India; 5 Department of Bioscience and Biotechnology, Konkuk University, Seoul, 05029 Republic of Korea; 6 Department of Public Health, Graduate School of Public Health, Seoul National University, Seoul, 08826 Republic of Korea

**Keywords:** Balanced Korean diet, Western diet, Oxidative stress, Serum biomarkers, Urine metabolomics, LC–MS/MS, Biochemistry, Biological techniques, Biomarkers

## Abstract

Dietary biomarkers in urine remain elusive when evaluating diet-induced oxidative stress and inflammation. In our previous study, we conducted a randomized controlled crossover trial to compare the short-term (4-weeks) effects of the balanced Korean diet (BKD) with Western diets, including the 2010 dietary guidelines for Americans (2010 DGA) and typical American diet (TAD), on various metabolic indices in obese Korean adults. Building on this work, the current research focuses on the impact of these dietary interventions on oxidative stress (d-ROMs and BAP) and inflammation (CRP, TNF-α, IL-6, IL-1β, MCP-1) biomarkers in serum, and the concurrent urine metabolomes. Each dietary regimen was in silico and experimentally examined for their antioxidant levels using ABTS, DPPH, and FRAP assays, as well as total flavonoid (TFC) and total phenolic (TPC) contents. We assessed post-intervention variations in oxidative stress and inflammation biomarkers in serum, as well as the urine metabolite profiles for the participants (*n* = 48, average age: 41 years). Antioxidant contents and associated total antioxidant capacity (TAC) were significantly higher for the recommended diets (BKD and 2010 DGA) compared to TAD (*p* < 0.05). Butanol extracts from recommended diets (BKD and 2010 DGA) showed significantly higher antioxidant activity compared to TAD in ABTS (*p* < 0.01), DPPH, and FRAP (*p* < 0.05) assays. Consistent results were observed in total phenolic and flavonoid contents, mirroring their respective antioxidant activities. Following the intervention period, oxidative stress & inflammation markers in serum varied marginally, however, the urine metabolite profiles were clearly demarcated for the BKD and Western dietary groups (PC1 = 5.41%). For BKD group, the pre- and post-intervention urine metabolite profiles were clearly segregated (PLS2 = 2.93%). Compared to TAD, urine extracts from the recommended dietary group showed higher abundance of benzoic acid & phenolic derivatives (VIP > 0.7, *p* < 0.05). Metabolites associated with oxidative stress were observed higher in the urine samples from Western dietary groups compared to BKD. Urine metabolomics data delineated the post-intervention effects of three dietary interventions which corroborates the respective findings for their effects on metabolic indices.

## Introduction

Studies have shown that both the long- & short-term dietary interventions influence the oxidative stress and systemic chronic inflammations (SCI) in humans^1–4^. A higher imbalance in the ratio of free radicals to the antioxidant species either through nutritional or endogenous factors is considered as the oxidative stress, which influences the progression of inflammatory chronic diseases^5^. Healthy dietary preferences are key to manage chronic inflammatory diseases which roughly accounts for approximately 50% of all the annual deaths and disabilities worldwide^6^. Diet induced SCI is associated with various metabolic disorders including cardiovascular diseases (CVD), stroke, insulin resistance, obesity, and cancer, among others^7^. Different dietary regimens are studied worldwide toward achieving a sustainable health with particular emphasis on oxidative stress and associated SCI, and their impact on etiological factors. In addition to the endogenous factors, the diet induced oxidative stress play a pivotal role in maneuvering various pathophysiological conditions. In particular, the unbalanced consumption of macronutrients, especially the animal-derived fats, promote oxidative stress, and subsequent inflammation through promoting the release of pro-inflammatory cytokines^8,9^.

Though the effects of different dietary regimens on anthropometric parameters are well studied, their correlations with the oxidative stress biomarkers in serum and urine metabolomes are largely unknown. The effects of dietary intakes and their metabolic implications for oxidative stress are mostly examined using the serum samples. However, the non-invasive and low-cost nature of urinary sample collection coupled with the unprecedented advancements in the mass spectrometry (MS) and spectroscopy (NMR) platforms makes it ideal for dietary intervention studies. In recent years, urine metabolomics has increasingly been used to monitor the effects of human exposure to drugs, toxins, pathogens, and related pathophysiological states^10^. Hence, a comprehensive data integration correlating the quantitative urinary metabolite profile with the blood indices of oxidative stress and the associated anthropometric parameters may help indicate the real-time clinical outcomes of dietary intakes. Moreover, the respective data may be subjected to translational assessment of diet induced SCI and predicting its likely clinical implications. Hence, the exploratory urinary metabolomics towards probing the effects of varying dietary regimens and the associated metabolic markers of SCI can help predict and mitigate the early onset of chronic diseases.

The greater adherence to the balanced dietary recommendations lowers the risk of SCI pertaining to the higher antioxidant capacity of dietary components^11^. Though the health beneficiary functions of the balanced Korean diet (BKD) rich in dietary phytochemicals are largely acknowledged, its metabolomic implications are relatively unknown owing to the lack of randomized crossover longitudinal studies. A quintessential BKD is mainly composed of plant-derived components (whole grains, vegetables, fruits, and fermented beans) with relatively lesser proportions of animal-derived and processed foods. Previously, we have demonstrated that the BKD significantly improves the obesity-related metabolic risk factors and blood lipid profiles in participants from a randomized controlled trial^12^. The present study explored the effects of short-term dietary interventions of the BKD and Western diets in participants and correlated the untargeted urine metabolite profiles with oxidative stress biomarkers in serum.

## Results

### Characteristics of the three different dietary regimens (BKD, 2010 DGA, and TAD)

#### Nutrient composition

We estimated the macronutrient and micronutrient contents including the antioxidant vitamins and phenolic compounds for each study diet. Notwithstanding their equivalent calorific values (~ 2000 kcal/day), BKD was characterized by the significantly higher relative abundance of total carbohydrates (*p* < 0.01) with least relative abundance of fats (*p* < 0.01). On the other hand, both the 2010 DGA and TAD were characterized by relatively higher fat contents (TAD > 2010 DGA; *p* < 0.01). However, the protein contents in all three dietary regimens were marginally varied (Supplementary Fig. S.1).

**Figure 1:**
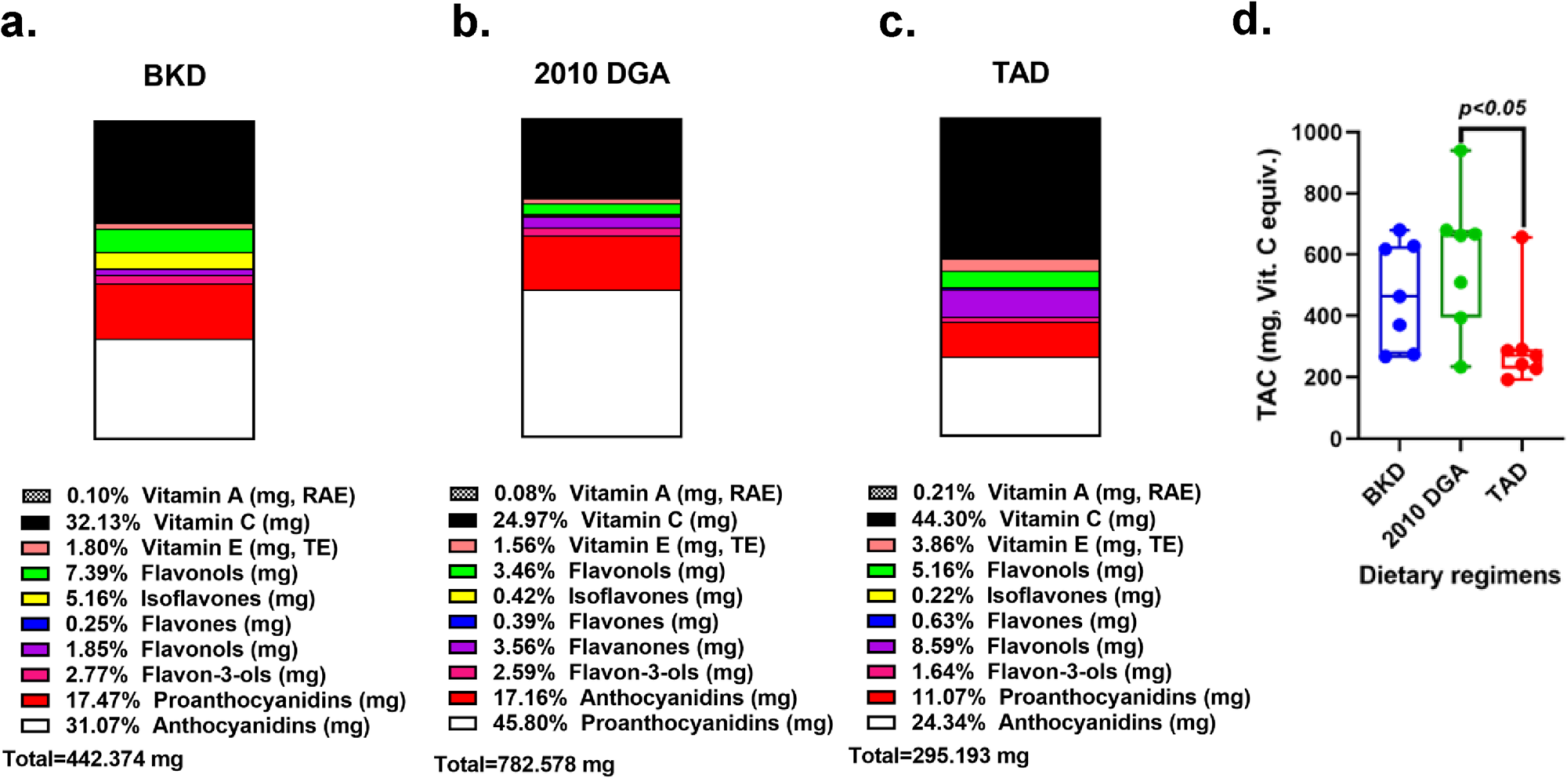
Stacked bar graph indicating micronutrient contents for the three dietary regimens used in the study; (**a**) BKD—balanced Korean diet, (**b**) 2010 DGA—dietary guidelines for Americans, and (**c**) TAD—typical American diet. The box and whisker plot show the varying levels of (**d**) total antioxidant capacity (TAC) estimated for each of the diet with their statistical significance were expressed based on the Tukey’s post-hoc test. These components were computed based on the corresponding theoretical values estimated for the food components in each dietary regimen provide four times a day per week. All three diets were designed to provide ~ 2000 kcal of energy per day from three meals.

For the micronutrients, vitamins and phenolic compounds were markedly varied among the three different dietary interventions (Fig. 1 and Supplementary table S.1). Most notably, vitamin A variants were lower in the BKD compared to the 2010 DGA and TAD, although the differences were not observed statistically significant. However, the vitamin A subtypes including α-carotene, β-carotene, β-cryptoxanthin, and lutein/zeaxanthin were relatively higher in the BKD and the 2010 DGA compared to TAD. Vitamin C contents were significantly higher in the 2010 DGA (*p* < 0.05), followed by the BKD and TAD, respectively. Similarly, vitamin E and its derivatives were also observed significantly higher for the western dietary regimens (2010 DGA > TAD) compared to the BKD (*p* < 0.01), except for their γ-tocopherol contents. Meanwhile, the BKD was characterized by the higher relative abundance of flavonols and isoflavones (*p* < 0.01), compared to the 2010 DGA and the TAD. On the other hand, the 2010 DGA was observed having higher relative levels of most phenolic compounds including flavones, flavanones, flavan-3-ols (*p* < 0.05), anthocyanidins, and proanthocyanidins (*p* < 0.05). Altogether, the dietary TAC was considerably higher in the 2010 DGA (*p* < 0.05) and the BKD, compared to the TAD.

#### Antioxidant activities

**Figure 2:**
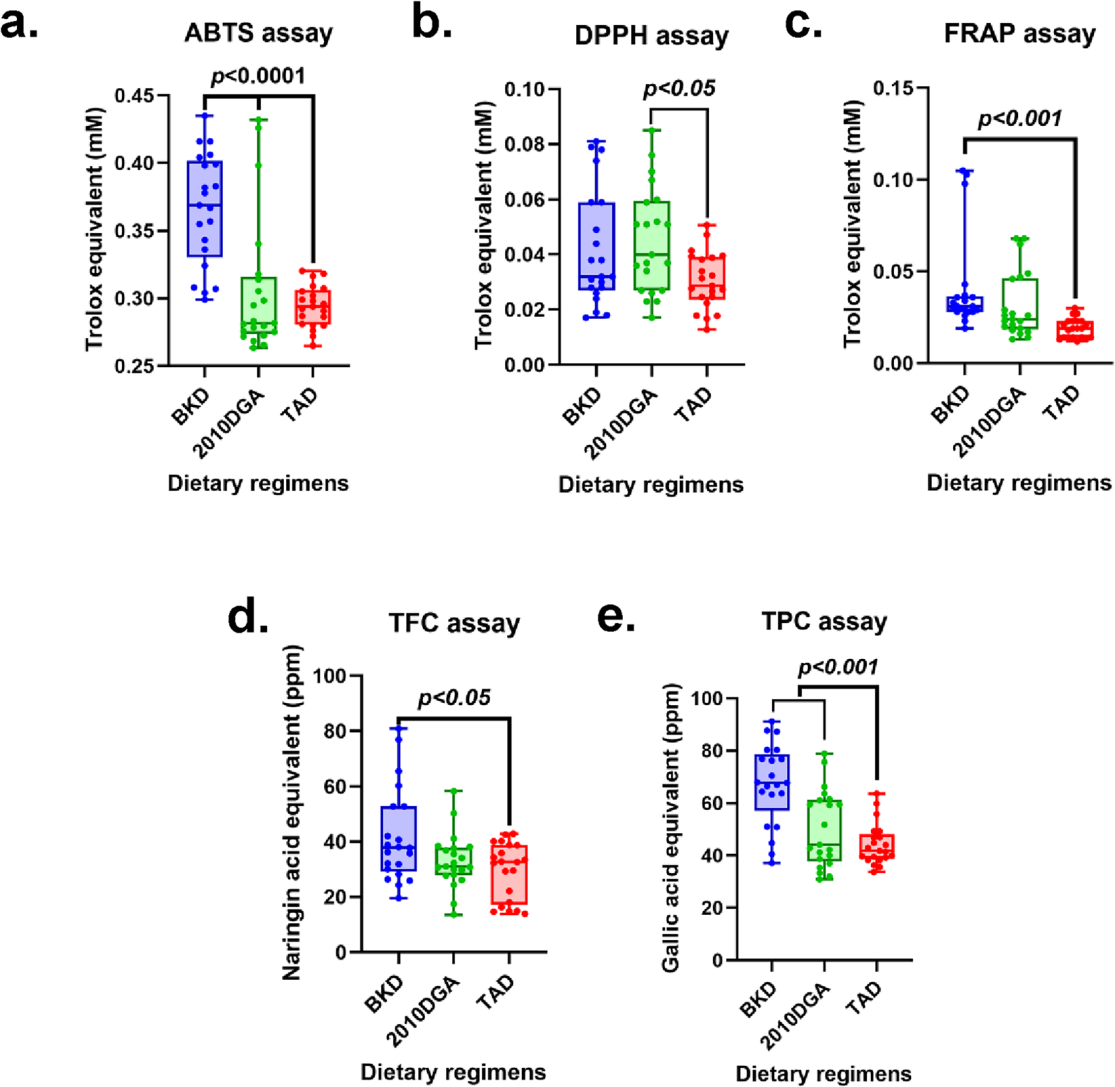
Box and whisker plots representing the varying antioxidant levels determined using the (**a**) ABTS, (**b**) DPPH, (**c**) FRAP assays, and the (**d**) total phenolic contents (TPC), and (**e**) total flavonoid contents (TFC) for the three different dietary regimen extracts. Plots a-e represents the bioactivities for butanol extracts of the corresponding food components from the week-long dietary menus given to the participants. The data shown here was recorded for 21 different meal samples representing 3 meals per day for 7 days under each dietary regimen. Abbreviations: BKD, balanced Korean diet; 2010 DGA, 2010 Dietary Guidelines for Americans; TAD, typical American diet. The data was subjected to Tukey’s post-hoc test to examine their statistical significance.

We examined the antioxidant levels in pooled sample menus representing the three different diets in this study. Notably, the butanol sample extracts from the BKD showed significantly higher antioxidant activities compared to the western diets (2010 DGA and TAD) in the ABTS (*p* < 0.0001) and FRAP (*p* < 0.001) assays (Fig. 2a and c). However, in the DPPH assay, the recommended western diet (2010 DGA) exhibited higher antioxidant bioactivities compared to the TAD (*p* < 0.05) as well as the BKD (Fig. 2b).

#### Total flavonoid and phenolic contents

The butanol extracts of BKD exhibited significantly higher total flavonoid contents (TFC) compared to the western diets (2010 DGA and TAD) at *p* < *0.05* (Fig. 2d). Similar patterns were observed for the total phenolic contents (TPC) in BKD, which were also significantly greater than those in both western diets (2010 DGA and TAD) at *p* < *0.001* (Fig. 2e).

### Characteristics of the study subjects

#### Baseline characteristics

Table 1 shows the baseline characteristics of the 48 study subjects before the onset of the dietary intervention trials. There were 25 males and 23 females’ participants enrolled in this study. Considering their baseline oxidative stress markers in serum, female participants had higher mean values of d-ROMs and lower mean values of BAP levels compared to male participants. For the inflammatory markers, the mean values of CRP and TNF-α levels were higher in female participants compared to males, while IL-6 and MCP-1 levels were relatively lower in females than in males. However, statistical significance for each parameter was not evaluated due to an insufficient number of subjects to verify the differences between men and women.

**Table 1:** Baseline characteristics of the study subjects before the onset of the trials.

Parameters	Total (n = 48)	Male (n = 25)	Female (n = 23)
Age (years)			
25–39	24	16	8
40–64	24	9	15
Monthly household income			
< 2 million KRW	9	6	3
2–6 million KRW	29	14	15
≥ 6 million KRW	10	5	5
Education level			
High School	11	2	9
College +	37	23	14
Alcohol consumption^1^			
No	12	3	9
Moderate	34	21	13
Heavy	2	1	1
Physical activity^2^			
No	39	21	18
Moderate	1	0	1
Vigorous	8	4	4
Oxidative stress indices (mean ± SD)			
d-ROMs (U. CARR.^3^)	381.25 ± 72.38	345.04 ± 54.01	420.61 ± 70.00
BAP (μmol/L)	2122.73 ± 267.59	2218.56 ± 224.39	2018.57 ± 276.15
Inflammatory indices (mean ± SD)			
CRP (mg/L)	2.06 ± 3.70	1.83 ± 3.88	2.31 ± 3.57
TNF-α (pg/mL)	2.18 ± 1.31	2.14 ± 1.24	2.23 ± 1.41
IL-6 (pg/mL)	1.85 ± 1.23	1.91 ± 1.40	1.78 ± 1.03
IL-1β (pg/mL)	0.06 ± 0.11	0.04 ± 0.04	0.08 ± 0.15
MCP-1 (pg/mL)	402.94 ± 130.65	418.06 ± 105.44	386.51 ± 154.26

KRW, Korean Won; SD, standard deviation; d-ROMs, diacron reactive oxygen metabolites; U. CARR., Carratelli Unit; BAP, biological antioxidant potential; CRP, C-reactive protein; TNF-α, tumor necrosis factor-α; IL-6, interleukin-6; IL-1β, interleukin-1β; MCP-1, monocyte chemoattractant protein-1.

^1^Moderate alcohol consumption was defined as drinking < 2 times/week, or drinking ≥ 2 times/week but < 7 cups/time for males and < 5 cups/time for female. Heavy alcohol consumption was defined as drinking ≥ 2 times/week and ≥ 7 cups/time for males and ≥ 5 cups/time for females.

^2^Moderate physical activity was defined as performing moderate-intensity physical activity for ≥ 30 min/time for ≥ 5 days/week. Vigorous physical activity was defined as performing vigorous intensity physical activity for ≥ 20 min/time for ≥ 3 days/week.

^3^U. CARR. = 0.08 mg/100 mL H_2_O_2_.

#### Oxidative stress and inflammatory biomarkers in serum

We observed that oxidative stress and inflammatory markers in serum samples exhibited slight variations among participants from the three dietary groups (Table 2). The recommended Korean diet (BKD) resulted in lower d-ROM levels compared to the recommended Western diet (2010 DGA). Conversely, BAP levels, which measure antioxidant capacity, were marginally higher in the 2010 DGA group than in the BKD group. Interestingly, the typical American diet (TAD) showed the lowest levels of both d-ROM and BAP. CRP levels, an inflammation marker, were lower in both the BKD and 2010 DGA groups compared to the TAD group. Furthermore, the 2010 DGA group exhibited the lowest levels of the inflammatory biomarker IL-6, followed by the TAD and BKD groups. Conversely, the TAD group showed the lowest levels of the inflammatory markers IL-1β and MCP-1 when compared to the BKD and 2010 DGA groups.

**Table 2:** Changes in serum oxidative stress and inflammatory indices after 4-weeks of dietary interventions^1^.

	BKD (n = 48)		2010 DGA (n = 48)		TAD (n = 48)	
	Mean ± SE	*p* value	Mean ± SE	*p* value	Mean ± SE	*p* value
d-ROMs (U. CARR.^2^)	7.02 ± 7.83	0.3722	10.65 ± 7.82	0.1767	4.04 ± 7.83	0.6069
BAP (μmol/L)	53.78 ± 45.04	0.2355	68.40 ± 45.02	0.1321	39.35 ± 45.05	0.3847
CRP (mg/L)	− 0.33 ± 0.52	0.5179	− 0.21 ± 0.52	0.6803	0.05 ± 0.52	0.9168
TNF-α (pg/mL)	− 0.14 ± 0.19	0.4453	0.17 ± 0.19	0.3736	0.01 ± 0.19	0.9404
IL-6 (pg/mL)	− 0.18 ± 0.20	0.3693	− 0.57 ± 0.20	0.0050	− 0.21 ± 0.20	0.3047
IL-1β (pg/mL)	0.08 ± 0.07	0.2138	0.04 ± 0.07	0.5225	− 0.02 ± 0.07	0.7735
MCP-1 (pg/mL)	− 47.54 ± 13.07	0.0005	− 38.56 ± 13.05	0.0040	− 54.12 ± 13.07	< 0.0001

BKD, balanced Korean diet; 2010 DGA, diet recommended by the 2010 dietary guidelines for Americans; TAD, typical American diet; SE, standard error; d-ROMs, diacron reactive oxygen metabolites; U. CARR., Carratelli Unit; BAP, biological antioxidant potential; CRP, C-reactive protein; TNF-α, tumor necrosis factor-α; IL-6, interleukin-6; IL-1β, interleukin-1β; MCP-1, monocyte chemoattractant protein-1.

^1^All analyses accounted for the crossover randomized control design using a mixed effect model adjusted for diet sequence effect and period effect.

^2^1 U. CARR. = 0.08 mg/100 mL H_2_O_2_.

Among these variations in serum biomarkers, statistically significant differences were observed only for IL-6 levels following consumption of the 2010 DGA (*p* = 0.0050). Additionally, post-intervention MCP-1 levels were significantly reduced in serum samples from participants in all three dietary groups (*p* = 0.0005 for BKD; *p* = 0.0040 for 2010 DGA; *p* < 0.0001 for TAD).

#### Urine metabolite profiles

**Figure 3:**
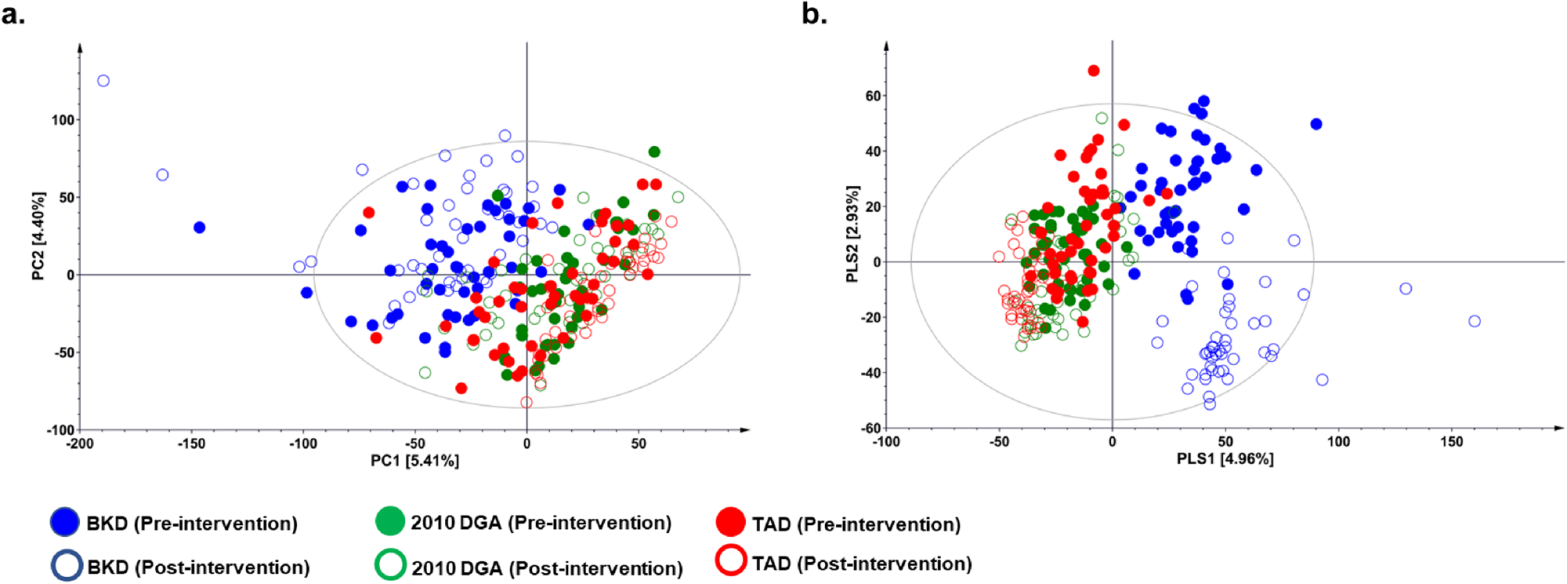
The (**a**) PCA and (**b**) PLS-DA score plots based on the negative ESI mode UHPLC-LTQ-Orbitrap-MS/MS datasets representing metabolite profiles for the pre- (filled circles) and post-intervention (empty circles) urine samples collected from the participants enrolled under three different dietary regimens. The urine sample datasets representing the participant enrolled in three different dietary regimens is indicated with color codes, Blue color: BKD (Balanced Korean diet), Green color: 2010 DGA (2010 Dietary Guidelines for Americans), and Red color: TAD (typical American diet).

**Figure 4:**
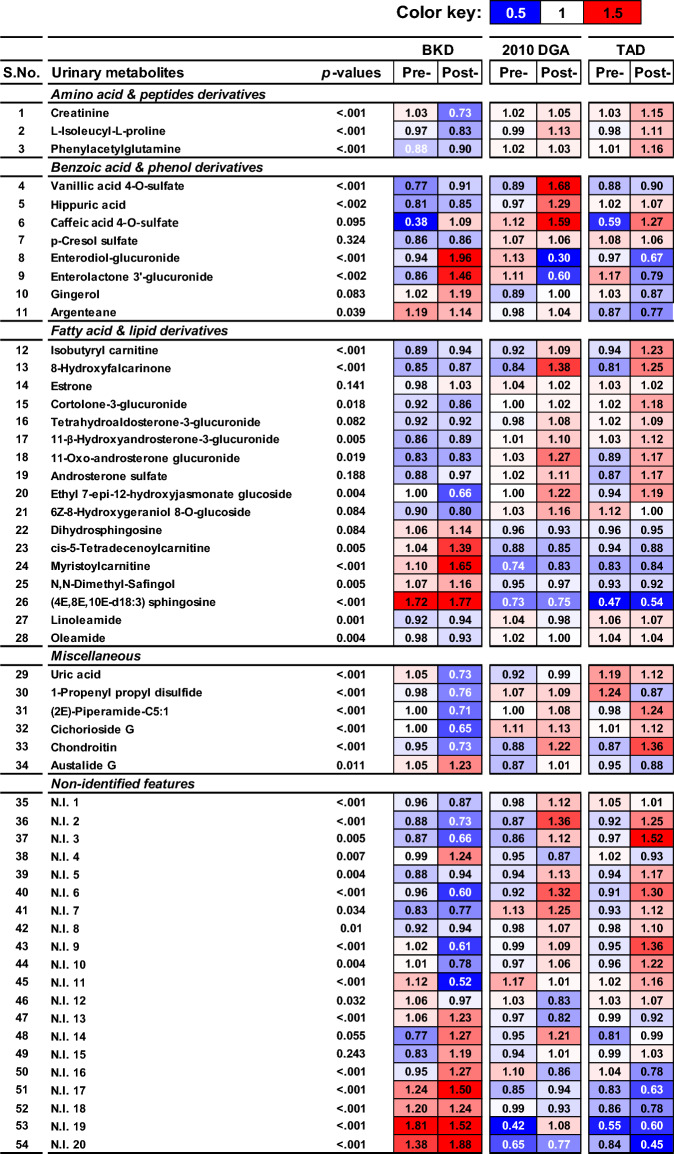
Heatmap representing the fold-change relative abundance of the significantly discriminant metabolites for the urine samples collected from the participants enrolled in BKD (Balanced Korean Diet), 2010 DGA (2010 Dietary Guidelines for Americans), and TAD (typical American diet). The sub-columns under each dietary group represent the pre- and post- intervention levels of each metabolite where the corresponding fold-change levels are indicated with the numerical values inside the corresponding box. The significantly discriminant metabolites were selected based on their VIP > 0.7 and *p* < 0.05 from the PLS-DA models build using the datasets acquired in the negative and positive ESI mode in UHPLC-LTQ-Orbitrap MS/MS.

Principal component analysis (PCA) score plot based on the negative ESI mode LC–MS datasets for the urine samples indicated a clear segregation between the study groups subjected to the BKD and western diets (2010 DGA and the TAD) across PC 1 (5.41%) with few outliers (Fig. 3). However, the datasets for pre- and post-intervention stages were not clearly segregated for each of the study group in the respective PCA plot. Supervised partial least squared—discriminant analysis (PLS-DA) score plot displayed a clearly clustered pattern for the BKD and western dietary regimens (2010 DGA and TAD) across PLS 1 (4.96%) whereas the respective pre- and post-intervention sample datasets were segregated across PLS 2 (2.93%). PLS-DA model was validated against the data overfitting with *Q*^2^ = 0.69, while its predictive accuracy was verified with *R*^*2*^*X* (0.28) and *R*^*2*^*Y* (0.96). In addition, Similar multivariate statistical patterns were evident for the urine metabolite profiles in positive ion mode (Supplementary Fig. S.2). We conducted cross-validation analysis to ensure the robustness of the described PLS-DA models and prevent the potential data overfitting (Supplementary Fig. S.3). Herein, the metabolite profiling data substantiates a multiparametric variability among the urine metabolite profiles highlighting the effects of short-term dietary interventions. Based on the PLS-DA model, we selected 54 significantly discriminant metabolites (VIP > 0.7, *p* < 0.05) which contribute maximally toward the observed variance among the urine metabolite profiles from the three dietary intervention groups. Among the selected candidates, 34 metabolites were putatively characterized mainly as the derivatives of amino acids & peptides (3), benzoic acid & phenolic (8), fatty acid & lipids (17), and six miscellaneous compounds (Fig. 4). The remaining 20 features were not characterized and labelled as non-identified (N.I). The LC–MS characteristics and the raw data peak intensities for each of the significantly discriminant metabolites are shown in supplementary data 1.

We showed the fold-change relative abundance of the significantly discriminant metabolites using the tabular heatmap (Fig. 4). Most notably, the urine metabolite profile from participants subjected to the 2010 DGA and TAD displayed significantly higher post-intervention abundance of the amino acid & peptide derivatives (creatinine, L-isoleucyl-L-proline, and phenylacetylglutamine) compared to those enrolled for BKD. Compared to the western dietary regimens, participants enrolled in BKD displayed higher relative abundance of most benzoic acid & phenolic derivatives (enterodiol-glucuronide, enterolactone-3’-glucuronide, and gingerol) except argenteane in corresponding urinary extracts. Notably, the participant enrolled with 2010 DGA group also displayed a higher urinary abundance of few plant-derived phenolics including vanillic acid 4-O-sulfate, hippuric acid, and caffeic acid 4-O-sulfate. Most benzoic acid & phenolic derivatives were least abundant in the urinary samples from the participants in TAD group. Considering the fatty acid & lipid derivatives, a higher relative abundance of most steroidal glycosides was evident in the post-intervention urine samples representing the participants in TAD group followed by 2010 DGA. However, the acylcarnitine compounds (cis-5-tetradecenoylcarnitine and myristoylcarnitine) were significantly higher in the post- intervention urinary samples from the BKD participants. Most sphingolipid derivatives were significantly higher in the post-intervention urinary samples from BKD groups. A fatty acid alcohol 8-hydroxyfalcarinone was significantly higher in post- intervention urine samples from the western dietary (2010 DGA and TAD) groups. Miscellaneous categories of metabolites including uric acid, propenyl propyl disulfide, piperamide-C5:1, cichorioside G, and chondroitin were more abundant in the post- intervention urine samples for the participant groups enrolled in western dietary regimens compared to those with BKD. In contrast, Austalide G, a metabolite derived from the soy-food fermentation, was significantly higher in the post- intervention urine samples from the BKD group. Considering the non-identified (N.I) features, post- intervention urinary samples from the participants from enrolled with western dietary regimens displayed higher relative abundance of features ranging N.I.1—N.I.10, while those from the BKD groups were more abundant in N.I. 13–20.

### Bivariate correlations between urinary metabolites and serum biomarkers

**Figure 5:**
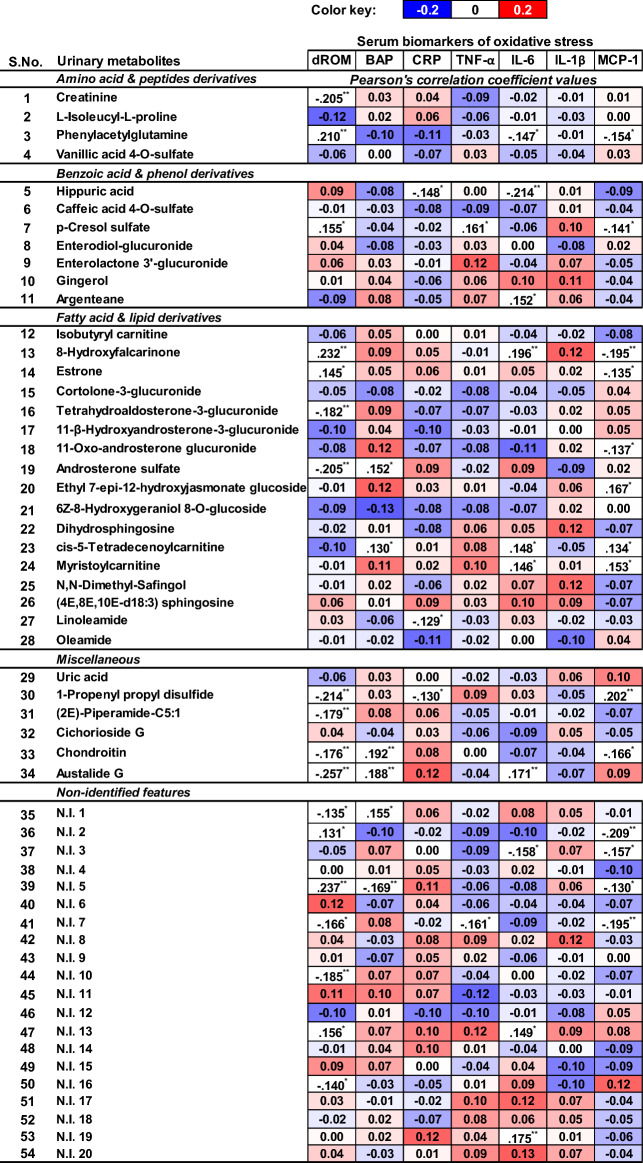
Heatmap showing the Pearson's correlations between the significantly discriminant (VIP > 0.7, *p* < 0.05) urinary metabolites and the serum biomarkers of oxidative stress & inflammation representing the pre- and post-intervention samples from participants enrolled in dietary trials. The level of significance (2-tailed) for these correlations are indicated with **0.01 and *0.05.

Bivariate Pearson's correlations between urinary metabolites and serum biomarkers of oxidative stress and inflammation revealed their patterns and linearity following the dietary interventions (Fig. 5). Positive correlations (0 < r ≤ 0.4) were observed between certain amino acid and peptide derivatives in urine, such as L-Isoleucyl-L-Proline and Phenylacetylglutamine, and serum biomarkers like BAP and CRP (Fig. 5). Notably, serum MCP-1 showed weak positive correlations (0 < r ≤ 0.4) of varying strengths with all amino acid and peptide derivatives except phenylacetylglutamine. Most benzoic acid and phenol-derived metabolites exhibited weak positive correlations (0 < r ≤ 0.4) with serum levels of dROM (except argenteane), TNF-α (except caffeic acid 4-O-sulfate), and IL-1β (except enterodiol-glucuronide). Conversely, weak negative correlations (0 > r ≥ − 0.4) were evident between most urinary metabolites and serum biomarkers of inflammation like CRP, IL-6, and MCP-1, with some exceptions. The relative abundance of most fatty acid and lipid derivatives in urine showed weak positive correlations (0 < r ≤ 0.4) with serum BAP and inflammation biomarkers such as TNF-α, IL-6, IL-1β, and MCP-1, with some exceptions. Selected fatty acid derivatives in urine, including 8-hydroxyfalacrinone, esterone, androsterone sulfate, and myristoylcarnitine, exhibited weak positive bivariate correlations with serum dROM and MCP-1, with some exceptions. Additionally, selected compounds such as piperamide C5:1, cichorioside G, chondroitin, and austalide G displayed positive correlations with the inflammatory biomarker CRP in serum samples. Notably, most of the inflammatory biomarkers in serum showed either weak negative (0 > r ≥ − 0.4) or neutral correlations with these compounds.

Furthermore, most non-identified (N.I) metabolites in urine displayed weak positive or no correlations with serum biomarkers such as dROM, BAP, CRP, TNF-α (N.I. 16—N.I. 20), IL-6 (N.I. 15—N.I. 20), and IL-1β (N.I. 17—N.I. 20).

## Discussion

Metabolic disorders related to oxidative stress and SCI can be effectively managed using the evidence-based dietary recommendations in which the higher abundance of antioxidant compounds ought to assuage cellular injuries mediated by free radicals. Herein, we examined the comparative effects of the short-term dietary interventions involving BKD, 2010 DGA, and TAD on oxidative stress & inflammatory indices among the Korean adults with obesity. Further, we examined the urinary biomarkers to examine the health effects of these dietary interventions using the untargeted metabolomics approach. Previously, we have shown that BKD effectively improves the three major metabolic indices including the BMI, body fat percent, and blood lipid profiles^12^. We assume that such different outcomes after 4-weeks of dietary interventions are associated to their varying antioxidant compositions which are primarily derived from the phytochemical components. Dietary intakes of phytochemicals with antioxidant functions have been extensively reported to be inversely linked with metabolic disorders in several epidemiological studies^13–19^.

### Recommended diets (BKD and 2010 DGA) provided more antioxidants than TAD

Recommended dietary regimens (BKD and 2010 DGA) had larger proportions of fruits, vegetables, legumes, whole grains & cereals, nuts, and unprocessed dairy with relatively lesser proportions of meat and poultry. However, a relatively larger portion of TAD included refined grains, processed meat, canned foods, and processed dairy ingredients^12^. Considering the varying levels of antioxidants in each of the dietary regimens, we examined the contents of antioxidant vitamins and polyphenols in each dietary regimen using in silico methods (Fig. 1). Higher relative abundance of vitamin A subtypes was evident for the recommended diets (BKD and 2010 DGA) which can be associated with their higher antioxidant potentials compared to TAD. Vitamin A and its various subtypes functions as provitamin carotenoid derivatives which inhibit the production of pro-inflammatory cytokines, prostaglandin E2, and nitric oxide in the body, and hence alleviate oxidative stress^20,21^. Higher titers of vitamin C and most vitamin E subtypes in recommended diets can be linked with their antioxidant potentials owing to their inhibitory activities against the production of pro-inflammatory cytokines like IL-4, IL-5, and IL-13^22,23^. Phenolic compounds are often touted as nutraceuticals owing to their ROS-scavenging effects and are reported from a variety of plant-derived foods including herbs, vegetables, fruits, spices, and associated beverages^24^. BKD was characterized with higher relative levels of most flavonols (quercetin, kaempferol, isorhamnetin) except myricetin (2010 DGA > BKD) which can be attributed to a variety of plant-derived dietary menu components including onions, apples, and various green leaf and cruciferous family vegetables^25^. Moreover, higher levels of isoflavones (daidzein, genistein, and glycitein) in the BKD compared to western diets is ascribed to the fermented soy-foods in Korean cuisines. Most other polyphenols (flavones, flavanones, flavan-3-ols, and anthocyanidins) were observed relatively higher in recommended diets (BKD and 2010 DGA) compared to TAD owing to the higher proportions of the plant-derived components^26^. We retrospectively examined the in vitro antioxidant levels for the weekly menus provided in each dietary regimen to the study participants. Intriguingly, the sample extracts for BKD and 2010 DGA rich in plant-derived nutrients displayed significantly higher antioxidant activities as well as total phenolic (TPC) and total flavonoid (TFC) contents compared to TAD (Fig. 2). Hence, *the in* vitro antioxidant activities and TPC & TFC levels for each dietary regimen extracts substantiated their *in-silico* micronutrient compositions.

### Short-term dietary interventions had minimal impact on serum biomarkers of oxidative stress and inflammation

The analysis of serum biomarkers for oxidative stress and inflammation showed varying effects of the three different dietary interventions. Considering the effects of dietary interventions in serum biomarkers of oxidative stress & inflammation, MCP-1 levels of the participants significantly declined after intervention periods with all three diets suggesting their beneficial effects on reducing inflammation under controlled conditions. Notably, 2010 DGA was also found to have an ameliorating effect on serum IL-6 levels compared to BKD and TAD groups. The levels of oxidative stress markers, d-ROMs, and antioxidant capacity marker, BAP, showed some differences across the dietary groups with BKD showed reduced oxidative stress with lower d-ROM levels compared to the recommended Western diet (2010 DGA), which had slightly higher BAP levels indicating better antioxidant capacity. However, the TAD group displayed the lowest d-ROM and BAP levels, suggesting the greatest reduction in oxidative stress but the lowest antioxidant capacity among the three dietary groups. The other inflammation markers (CRP, TNF-α, IL-6, and IL-1β) varied among the participants following the 4-weeks of dietary interventions, though none of these variations were statistically significant.

Here, we hypothesize that data misinterpretation might occur, especially since the western diets like TAD and 2010 DGA could potentially be more effective for participants who had been consuming a relatively unhealthier diet before joining the trial or during the 2-weeks washout periods. This consideration is important given that each dietary regimen had equivalent caloric values and balanced macronutrient compositions with controlled serving portions. However, the persistent and long-term carry-over effects of participants' usual dietary habits cannot be entirely excluded. To address this limitation in interpreting the fragmented results of short-term dietary interventions, we investigated the complementary changes in the participants' urinary metabolite profiles.

### Urine metabolomics unraveled subtle effects of short-term dietary interventions

Untargeted urine metabolomics data suggested a marked variation in the baseline metabolic fingerprints of the participants enrolled in Western (2010 DGA and TAD) and Korean (BKD) diets. We analyzed the significantly discriminant metabolites with known associations with the characteristic dietary intake and physiological functions linked with oxidative stress. Of the 54 significantly discriminant metabolites, 34 metabolites were identified while 20 remained non-identified, and therefore, we limited our discussion on the likely implications of the characterized metabolites. Participants subjected to western dietary regimens displayed higher urinary abundance of the amino acid & peptide derivatives compared to the BKD group. Especially, the peptides including creatinine and L-isoleucyl-L-Proline are often correlated with an increased dietary intake of red meat or the endogenous catabolism of proteins & muscle turnover in humans^27,28^. Higher urinary titers of phenylacetylglutamine are often linked with heightened ROS generation and metabolic disorder including obesity^29,30^.

Benzoic acid & polyphenol derivatives were relatively higher in the urine extracts from the participants enrolled with the recommended dietary regimens (BKD and 2010 DGA) compared to those with TAD. Higher abundance of phytoestrogen derivatives including the lignans (enterodiol-glucuronide and enterolactone 3’-glucuronide) in urine are the known biomarkers of whole grain (barley, rye, wheat) consumption and signifies their anti-inflammatory functions. In addition, flaxseeds, nuts, legumes, and sesame seeds also contain the considerably high proportions of dietary lignans which constitute an important portion of the traditional Korean diet as well as BKD^31^. In addition, the higher post- intervention levels of gingerol (a methoxyphenol compound) in urinary samples from the BKD group is ascribed to the introduction of herbs and spices including ginger in the diet. Gingerol is known for its antioxidant functions through inhibiting the release of pro-inflammatory cytokines in the blood^32^. We observed weak positive correlations between the polyphenol abundance in urine and the pro-inflammatory cytokines (TNF-α and IL-6) in serum samples from BKD enrolled participants which suggest their possible role in inflammatory response (Fig. 5). Participants subjected to the 2010 DGA showed higher urinary abundance of phenolic derivatives including vanillic acid 4-O sulfate, hippuric acid, and caffeic acid 4-O-sulfate which can be linked with the consumption of plant-derived components and whole grains. Hippuric acid levels in urine indicate their positive effects on antioxidant enzyme systems including CoQ (Co-enzyme Q10) and β-carotene in plasma. However, hippuric acid influences the inhibition of the endogenous antioxidative systems in humans which include Nrf2, thioredoxin, and superoxide dismutase, suggesting a balance between the blood and urinary levels of hippuric acid^33^.

Urinary abundance of fatty acid & lipid derivatives including most steroidal glycoside, acyl-carnitines, sterols, and terpene glycosides in the study group subjected to western diets (2010 DGA and TAD) were higher than those provided with BKD which can again be attributed to their different dietary compositions. Notably, elevated levels of urinary isobutyryl-L-carnitine and estrone (steroid lipid) are associated with higher consumption of red meat & animal products which constitute a major portion of western diets (www.foodb.ca). These lipid derivatives are known to induce oxidative stress through promoting the fatty acid oxidation and/or ROS generation in body^34,35^. Higher abundance of most steroidal- and terpene- glycosides in the urinary samples from participants enrolled with western diets suggest their higher dietary compositions and in situ biotransformation. Most notably, cortolone-3-glucuronide, tetrahydroaldosterone-3-glucuronide, 11-β-hydroxyandosterone-3-glucuronide, ethyl-7-epi-12-hydroxyjasmonate glucoside, and androsterone sulfate are produced by the endocrine transformation of food-derived nutrients. Most fatty acid & lipid derivatives in urine samples displayed weak positive correlations with pro-inflammatory cytokines (TNF-α and IL-1β) which suggest their role in oxidative stress (Fig. 5). Urine samples from the participants enrolled in BKD were more abundant in acylcarnitine compounds (cis-5-tetradecenoylcarnitine and myristoylcarnitine) associated with fatty acid oxidation and ROS generation^36,37^. However, higher relative levels of sphingolipids in post- intervention urine samples from BKD group suggests their ameliorating effects against obesity related malfunctions^38^.

Elevated levels of uric acid in body, an endogenous antioxidant metabolite and a ROS-scavenger, indicate the systematic oxidative stress in humans^39^. As we enrolled the healthy participants in this study and subjected them to the short-term dietary interventions, the higher urinary levels of uric acid indicate that oxidative stress build-up following the course of western dietary (TAD > 2010 DGA) interventions, and not the BKD (Fig. 5). Remaining metabolites including organic disulfide, piperamide, and cichorioside are the biomarkers of respective plant-derived food sources including onions, peppers & herbs, chicory, and endives served in all dietary regimens at various proportions (www.foodb.ca). Most of these compounds have a known antioxidant functions when taken as dietary component. Higher post- intervention abundance of Austalide G in urinary samples from participant subjected to BKD might be associated with unique inclusion of *Aspergillus* fermented food components. Austalide G is a polycyclic aromatic metabolite belonging to the chemical class of xanthenes which are reportedly produced by certain *Aspergillus* species however their health effects are largely unreported and hard to associate with oxidative stress.

Considering the strengths of this work, it’s arguably the first study focusing on the associations between the oxidative stress indices in serum, urinary metabolites, and dietary intervention comparing the BKD and western diets. The results may provide scientific evidence and rationale toward the establishment of novel dietary guidelines for the peoples with SCI and associated metabolic disorders. The present study involved crossover randomized controlled trials which could increase the statistical power and minimize the possible confounding effects even with the relatively small number of participants. However, we also acknowledge certain limitations such as the analysis of serum and urine samples following a brief period of storage. Though, the samples were quenched and stored under standard conditions (− 80 ˚C), we assume the degradation of certain unstable compounds. Further, the study diets used in the trials do not represent the typical dietetics patterns & habits of either the Koreans and/or Americans per se but rather designed based on the respective government guidelines and references.

In summary, the present study explores the associations between short-term dietary interventions and their effects on oxidative stress biomarkers in serum and metabolic signatures in urine. The plant-derived components (fruits, vegetables, legumes, nuts, and whole grains) in the BKD and 2010 DGA likely alleviated the oxidative stress in the participants, compared to those subjected to the TAD. However, varying degrees of animal-derived components (red meat, poultry, and processed dairy products) and processed foods in western dietary regimens also influenced oxidative stress and related biomarkers in body fluids. Overall, the nutritional quality of diets isn't solely reliant on including minimally processed whole food components and plant-derived antioxidants as observed for the recommended diets in this study. It's equally important to consider the proportions of ultra-processed animal-derived products, as they can significantly contribute to the oxidative stress. Diets recommended for their fine balance of healthy dietary components, such as the BKD and 2010 DGA, are considered better in this regard compared to the TAD. Besides perceived complications involving sample preparations and data analysis, we believe that the untargeted urine metabolomics approach use in this study can be leveraged to measure the effects of short-term dietary interventions and their physiological impacts in clinical trials or population studies.

## Materials and methods

### Study subjects

**Figure 6:**
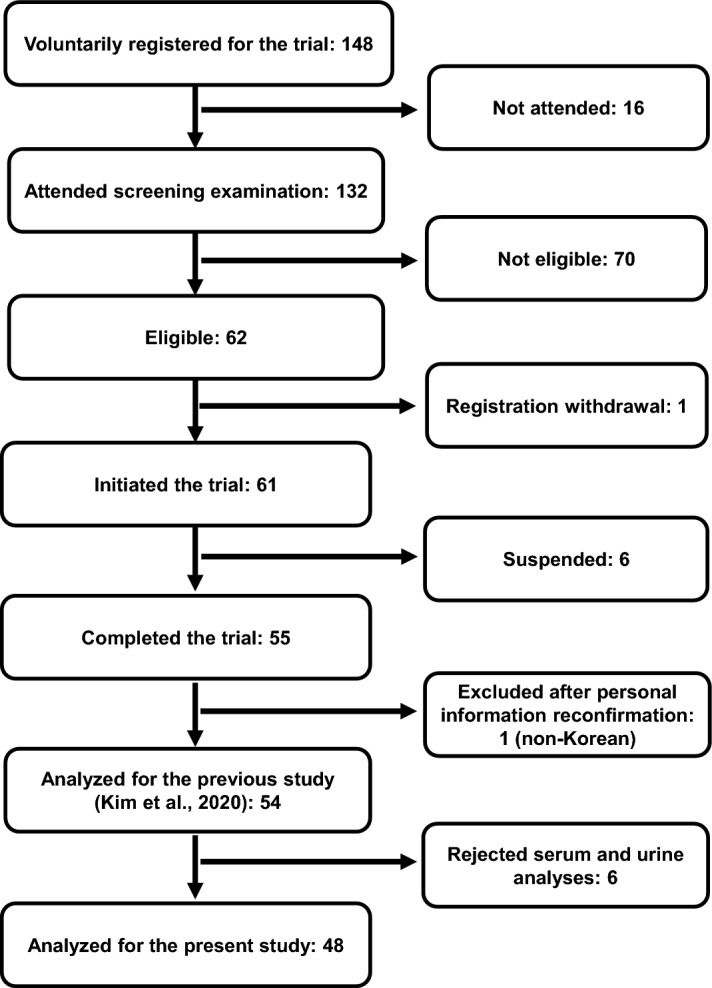
Flowchart illustrating the design of the study and participants at different stages of the dietary trials. The schematics was adopted from our previous study (ref.^36^).

As depicted in the participant flow chart (Fig. 6), all 148 participants affiliated to this study were voluntarily recruited through e-mail and poster advertisements as described previously by Kim et al.^12^ Anthropometric measurement, blood test, and face-to-face survey were initially performed for screening among 132 attendees. The inclusion criteria of the trial were Korean adults aged 25–65 years with body mass index (BMI) ≥ 23 kg/m^2^ and the blood low-density lipoprotein (LDL) cholesterol ≥ 120 mg/dL. Participants were excluded if they were smoking regularly and/or having the alcohol or substance abuse problems. We also excluded the participants which were consuming the prebiotics, probiotics, or antibiotics during the past 6 months before the onset of trials. Participants who reported a significant weight loss (≥ 10% of body weight) during the last 12 months before the trial period or those with any metabolic disorders including the cardiovascular diseases (CVD), diabetes, and kidney ailments were screened-out of the study. Sixty-one eligible individuals were selected for participation while only 54 of them completed the trials. The detailed information for this trial can be retrieved from our previous publications^12,40^.

Following the trial period, our research staffs responsible for maintaining the personal information contacted every subject to seek their approval for further analyses of oxidative stress and inflammatory biomarkers in serum samples, and their urine metabolite profiling. Of the total participants, 48 participants voluntarily approved their serum and urine analyses. We had confirmed using power analysis that a total of 30 participants were sufficient to detect a mean difference of LDL cholesterol, which is one of the major indices of blood lipid profile, in our previous study (α = 0.05, β = 0.20)^12^. All the steps in this trial were approved by the Institutional Review Board of Seoul National University (IRB No. 1506/002–014 & IRB No. 1805/003–010). The first date of study participant registration was 11/09/2015, and informed consents were obtained from all the participants. The whole processes of this study were performed in accordance with the relevant guidelines and regulations. This trial was registered at the clinical research registry, Clinical Research Information Service (CRIS) in Korea, which is the primary registry of the World Health Organization international clinical trial registry platform (registration No. KCT0002437; https://cris.nih.go.kr/cris/search/detailSearch.do?search_lang=E&focus=reset_12&search_page=L&pageSize=10&page=undefined&seq=8598&status=5&seq_group=8598).

This study was conducted following the CONSORT 2010 guidelines for reporting randomized controlled trials^41^.

### Design of study

Study design was adapted from the previous trial for dietary interventions conducted by Schroeder et al.^42^ with some modifications and described elsewhere in details^12,40^. It was a crossover randomized controlled trial with three intervention periods conducted over a period of from 2015 to 2017. Participants were stratified based on sex, BMI, and blood LDL cholesterol levels, and were randomly assigned into 6 groups according to the order of the three different dietary patterns. This included dietary regimens with balanced Korean diet (BKD), diet recommended by the 2010 Dietary Guidelines for Americans (2010 DGA), and typical American diet (TAD) for 4 weeks each. During the intervention periods, the subjects had to consume only the dishes provided in the study diets. The participants visited the research institute to have breakfast meals during weekdays under supervision of the research staffs, while lunch and dinner of weekday meals and whole meals during weekends or holidays were packed with cooler bags. The participants were asked to report to the chief research staff with pictures whether they consumed the whole meals provided every day. If there was any leftover, the research staffs estimated its calories based on the pictures taken by the participants; and the compliance was calculated by subtracting the calories of leftover from the total calories provided. The mean compliance rates of each dietary regimen were as follows: 98.1% for BKD, 97.8% for 2010DGA, and 98.1% for TAD^12^.

For all the participants, alcohol consumption was prohibited and maintaining the usual physical activity levels was strongly recommended to minimize the possible confounding effects. Between each intervention period, the subjects had a 2-week interval as washout period in which they were allowed to have their usual diets & lifestyle. Information on the usual diets of each participant at baseline and during the washout period were collected based on non-consecutive three-day diet record method, and their nutrient intakes were estimated using the CAN-Pro 5.0 (Computer Aided Nutritional analysis program 5.0, The Korean Nutrition Society, Seoul, Korea)^12^. At the beginning and the end of each intervention, the participants received physical examinations following a fasting period (≥ 8 h) and provided the first morning urine samples. Metabolic indices including the BMI, body fat percent, waist circumference, blood pressure, blood triglyceride & cholesterol, blood glucose, and blood insulin levels were obtained from anthropometric measurement and blood test. We have previously reported the effects of these dietary regimens on the above-mentioned metabolic indices of the participants^12^.

### Study diets

Details for dietary regimens including the BKD, 2010 DGA, and TAD and their components are described previously by Kim et al.^12^ and Shin et al.^40^. Each standardized dietary regimen was designed to supply 2000 kcal/day including the whole meals of breakfast, lunch, supper, and snack on a 7-day cycle. We have reported the calorific values for different nutritional components in each dietary regimen using the CAN-Pro 5.0 (Computer Aided Nutritional analysis program 5.0, The Korean Nutrition Society, Seoul, Korea) with each component analyzed according to the protocols from the Korean Food Standards Codex^12^. Each subject’s serving size was determined by the individual estimated energy requirement that was calculated based on the participant’s information for sex, age, body weight, height, and physical activity level using the formulas established by the Dietary Reference Intakes for Koreans (KDRI)^43^.

The BKD was developed based on the food guidance of the KDRIs and the dietary guidelines for Korean adults published by the Ministry of Health and Welfare, Republic of Korea^43–45^. The ratios of the energy supply from macronutrients in BKD were as follows: 60–65% from carbohydrate, 20–25% from fat, and 15% from protein. It included multi-grain rice, soup, 120 g/day of kimchi, and side dishes containing relatively large amounts of vegetables and legume products with 15 g/day of fermented ingredients such as red pepper paste and soybean paste. The 2010 DGA was developed based on the sample menus recommended by the 2010 Dietary Guidelines for Americans issued by the US Department of Agriculture (USDA)^46^. The proportion of the energy supply from the carbohydrate was 55%, whereas those from the fat and protein were 30% and 15%, respectively. It contained whole grains, vegetables, fruits, lean meat, seafood, and skim milk. The TAD was developed based on the data from the ‘What We Eat in America’ published by the National Health and Nutrition Examination Survey, 2001–2004^47^. The ratios of energy supply from macronutrients were as follows: 50% from carbohydrate, 35% from fat, and 15% from protein. It was mainly consisted of refined grains and processed foods with relatively small amounts of vegetables, fruits, and lean meat. The sample menus for each study are introduced in our previous study^12^.

### Antioxidant contents for the study diets

To estimate the antioxidant contents and dietary TAC (vitamin C equivalents) for the three study diets, we associated the recipes of each regimen to the databases of antioxidant capacity, antioxidant vitamins, and flavonoid contents available for commonly consumed Korean foods^48–50^. The following antioxidants components of the study diets were included in the databases: retinol, carotenoids (α-carotene, β-carotene, lycopene, β-cryptoxanthin, lutein/zeaxanthin), vitamin C, tocopherols (α-tocopherol, β-tocopherol, γ-tocopherol, δ-tocopherol), flavonols (quercetin, kaempferol, isorhamnetin, myricetin), isoflavones (daidzein, genistein, glycitein), flavones (apigenin, luteolin), flavanones (eriodictyol, hesperetin, naringenin), flavan-3-ols (catechin, epicatechin, epigallocatechin), anthocyanidins (cyanidin, delphinidin, pelargonidin, malvidin, peonidin, petunidin), and proanthocyanidins (dimers, trimers, 4–6mers, 7–10mers, 10 + polymers). Vitamin A and vitamin E levels were calculated in retinol activity equivalent (RAE) and α-tocopherol equivalent (α-TE), respectively, using the following formulas:

1 $$ {\text{RAE}},\;\upmu {\text{g}} = \left( {{\text{retinol}},\upmu {\text{g}} } \right) + \frac{{\upbeta \;{\text{carotene}},\upmu {\text{g}}}}{12} + \frac{{\upalpha \;{\text{carotene}},\upmu {\text{g}}}}{24} + \frac{{\upbeta \;{\text{cryptoxanthin}},\upmu {\text{g}}}}{24} $$

2 $$ \upalpha {-}{\text{TE}}, {\text{mg}} = \left( {\upalpha \;{\text{ tocopherol}}, {\text{mg}}} \right) + \left( {\upbeta \;{\text{tocopherol}} \times 0.5,{\text{mg}}} \right) + \left( {\upgamma \;{\text{ tocopherol}} \times 0.1,{\text{mg}}} \right) + \left( {\updelta \;{\text{tocopherol}} \times 0.03, {\text{mg}}} \right) $$

The experimental analyses of the total phenolic contents (TPC), the total flavonoid contents (TFC), and overall antioxidant levels of the study diets were conducted using the methods adapted from Jun et al.^51^ Daily menus of each of the study diet were blended, homogenized, and stored immediately at − 80℃ until analyses. Prior to the extraction, the samples were lyophilized using a freeze dryer (Bondiro, Ilshin Lab Co., Gyeonggi-do, Korea). Eight grams of dried samples from each dietary menu were subjected to initial extraction with 70% ethanol (1:1, w/v) by incubating them at 300 rpm for 1 h at 24 °C. Subsequently, the samples were centrifuged (8000 rpm for 10 min at 4 °C) and the resulting supernatants were filtered, and further dried using a speed vacuum concentrator (Hanil Scientific, Korea). The ethanolic extracts were then dissolved in water (1:1, w/v) and partitioned with butanol (1:1, v/v). The supernatant butanol fractions were isolated, dried using a speed vacuum concentrator, and subsequently reconstituted again to attain a final concentration of 10 mg/mL, before conducting bioactivity assays. TPC, TFC, and the antioxidant levels (ABTS assay, DPPH assay, and FRAP assay) for the dietary sample extracts were determined using the method adapted from our previous study by Lee et al.^52^.

### Analyses of oxidative stress and inflammatory markers in serum samples

Serum samples were collected on the days of physical examination and were immediately stored at − 80℃ until analyses. Oxidative stress biomarkers in serum including d-ROMs (diacron reactive oxygen metabolites) and BAP (biological antioxidant potential) were measured using an automatic chemistry analyzer (Diacron International s.r.l., Grosetto, Italy). The levels of inflammatory biomarkers in serum including C-reactive protein (CRP), tumor necrosis factor-α (TNF-α), interleukin-6 (IL-6), interleukin-1β (IL-1β), and monocyte chemoattractant protein-1 (MCP-1) were determined using Multiplex Luminex Assay (R&D Systems, Minneapolis, MN, USA).

### Metabolite profiling of urine samples

Pooled urine samples (400 μL) were extracted with 1 mL of absolute methanol and centrifuged at 13,000 rpm for 10 min at 4˚C. Supernatants were collected and dried using speed vacuum concentrator. Dry sample extracts were reconstituted in methanol at the concentration of 50 mg/mL and passed through 0.2 μm filter prior to the untargeted LC–MS analysis. Samples were on run on the UHPLC-LTQ-Orbitrap-MS/MS system coupled with Vanquish binary pump H system (Thermo Fisher Scientific, Waltham, Massachusetts, USA). Reverse phase chromatographic separation of metabolites was performed on Phenomenex KINETEX® C18 column (100 mm × 2.1 mm, 1.7 μm particle size; Torrance, CA, USA). The mobile phase composed of water (solvent A) and acetonitrile (solvent B) with 0.1% formic acid in each. The 14 min gradient run program commenced with 5% solvent B for 1 min followed by its linear increase to 100% in next 9 min, maintained for 1 min, and re-equilibrated to initial condition (5% solvent B) in the final 3 min. The chromatographic run program maintained a constant flow rate of 0.3 mL/min with a sample injection volume of 5 μL and the column temperature at 40 °C. The tandem MS was performed on LTQ-Orbitrap-Velos Pro with ion-trap (IT) MS and heated ESI or HESI-II probe (Thermo Fisher Scientific). The MS parameters were fixed at probe heater temperature of 300 °C, capillary temperature of 350 °C, and the capillary voltages of 2.5 kV (− ESI) and 3.7 kV (+ ESI). The samples were analyzed over a mass range (m/z) ranging from 150–1000 under both positive and negative ESI modes.

### Data processing and multivariate statistical analyses

Changes in oxidative stress and inflammatory indices in the serum samples for each dietary intervention group were investigated using a ‘mixed effect model’ adjusted for the dietary sequences and the washing period, which accounted for the crossover design. Post hoc analysis using Tukey’s HSD (honest significant difference) test was performed to examine the differences between the diets. The analyses were conducted using SAS 9.4 (SAS Institute Inc., Cary, NC, USA). A two-sided *p* value < 0.05 was considered statistically significant.

The raw data files obtained from UHPLC-LTQ-Orbitrap-MS/MS system were converted to NetCDF (network Common Data Form) file formats. The converted files (*.cdf*) were pre-processed for peak list alignment, peak detection, retention time (RT), normalized peak intensities, and accurate masses comparing their full scan nominal mass using the MetAlign™ software. The aligned data were further subjected to multivariate analyses to evaluate the class-wise variance in datasets and determining the significantly discriminant metabolites (VIP > 0.7, *p* < 0.05) based on the PLS-DA model made with SIMCA-P + (version 12.0, Umetrics, Umea, Sweden). The heat map expressions for the metabolite levels and corresponding pairwise correlation (PASW statistics) were made on Microsoft Excel 2016.

### Metabolite annotations

The significantly discriminant features were classified based on the PLS-DA model for LC–MS datasets, and the metabolites were putatively identified based on their RT, mass to charge ratios (m/z), MS^n^ fragmentation patterns, and elemental compositions (error window < 10 ppm) with corresponding standards, *in house* libraries, associated web databases. The food derived and urinary metabolites were characterized using a variety of databases and associated literature sources, but all are represented together according to their levels at the beginning and the end of each intervention.

### Supplementary Information

Supplementary Information 1.

Supplementary Information 2.

## Data Availability

The datasets used and/or analysed during the current study available from the corresponding author on reasonable request.

## Abbreviations

BKD: Balanced Korean diet
2010 DGA: 2010 Dietary Guidelines for Americans
TAD: Typical American Diet
TAC: Total antioxidant capacity
SCI: Systemic chronic inflammations

## References

[1] Aleksandrova K, Koelman L, Rodrigues CE (2021). Dietary patterns and biomarkers of oxidative stress and inflammation: A systematic review of observational and intervention studies. Redox Biol..

[2] Xiao YL, Gong Y, Qi YJ, Shao ZM, Jiang YZ (2024). Effects of dietary intervention on human diseases: Molecular mechanisms and therapeutic potential. Signal Transduct. Target Ther..

[3] Koelman L, Rodrigues CE, Aleksandrova K (2022). Effects of dietary patterns on biomarkers of inflammation and immune responses: a systematic review and meta-analysis of randomized controlled trials. Adv Nutr..

[4] Kaushik AS, Strath LJ, Sorge RE (2020). Dietary interventions for treatment of chronic pain: Oxidative stress and inflammation. Pain Ther..

[5] Rupérez AI, Gil A, Aguilera CM (2014). Genetics of oxidative stress in obesity. Int. J. Mol. Sci..

[6] Furman D (2019). Chronic inflammation in the etiology of disease across the life span. Nat. Med..

[7] Zhong X (2017). Inflammatory potential of diet and risk of cardiovascular disease or mortality: A meta-analysis. Sci. Rep..

[8] de Souza RJ, Swain JF, Appel LJ, Sacks FM (2008). Alternatives for macronutrient intake and chronic disease: A comparison of the OmniHeart diets with popular diets and with dietary recommendations. Am. J. Clin. Nutr..

[9] Herieka M, Erridge C (2014). High-fat meal induced postprandial inflammation. Mol. Nutr. Food Res..

[10] Wishart DS (2016). Emerging applications of metabolomics in drug discovery and precision medicine. Nat. Rev. Drug Discov..

[11] Song Y, Joung H (2012). A traditional Korean dietary pattern and metabolic syndrome abnormalities. Nutr. Metab. Cardiovasc. Dis..

[12] Kim SA (2020). Effect of a balanced Korean diet on metabolic risk factors among overweight/obese Korean adults: A randomized controlled trial. Eur. J. Nutr..

[13] Son SY (2016). Metabolite fingerprinting, pathway analyses, and bioactivity correlations for plant species belonging to the Cornaceae, Fabaceae, and Rosaceae families. Plant Cell. Rep..

[14] Zamora-Ros R (2011). Estimation of the intake of anthocyanidins and their food sources in the European Prospective Investigation into Cancer and Nutrition (EPIC) study. Br. J. Nutr..

[15] Zhang YJ (2015). Antioxidant phytochemicals for the prevention and treatment of chronic diseases. Molecules.

[16] Bertoia ML (2016). Dietary flavonoid intake and weight maintenance: three prospective cohorts of 124,086 US men and women followed for up to 24 years. B. M. J..

[17] Quansah DY (2017). Associations of dietary antioxidants and risk of type 2 diabetes: Data from the 2007–2012 Korea National Health and Nutrition Examination Survey. Molecules.

[18] Dollerup OL (2018). A randomized placebo-controlled clinical trial of nicotinamide riboside in obese men: Safety, insulin-sensitivity, and lipid-mobilizing effects. Am. J. Clin. Nutr..

[19] Ham D (2019). Consumption of Korean foods with high flavonoid contents reduces the likelihood of having elevated C-reactive protein levels: Data from the 2015–2017 Korea National Health and Nutrition Examination Survey. Nutrients.

[20] Milani A, Basirnejad M, Shahbazi S, Bolhassani A (2017). Carotenoids: Biochemistry, pharmacology and treatment. Br. J. Pharmacol..

[21] Zhou L (2018). Protective role of β-carotene against oxidative stress and neuroinflammation in a rat model of spinal cord injury. Int. Immunopharmacol..

[22] Peh HY, Tan WS, Liao W, Wong WS (2016). Vitamin E therapy beyond cancer: Tocopherol versus tocotrienol. Pharmacol. Ther..

[23] Azzi A (2018). Many tocopherols, one vitamin E. Mol. Aspects. Med..

[24] Vamanu E (2019). Polyphenolic nutraceuticals to combat oxidative stress through microbiota modulation. Front. Pharmacol..

[25] Li Z (2018). Profiling of phenolic compounds and antioxidant activity of 12 cruciferous vegetables. Molecules.

[26] Speer H, D’Cunha NM, Alexopoulos NI, McKune AJ, Naumovski N (2020). Anthocyanins and human health—A focus on oxidative stress, inflammation and disease. Antioxidants.

[27] Cross AJ, Major JM, Sinha R (2011). Urinary biomarkers of meat consumption. Cancer Epidemiol. Biomarkers Prev..

[28] Pivovarova-Ramich O (2020). Effects of diets high in animal or plant protein on oxidative stress in individuals with type 2 diabetes: A randomized clinical trial. Redox Biol..

[29] Hasegawa S, Jao TM, Inagi R (2017). Dietary metabolites and chronic kidney disease. Nutrients.

[30] Yu HT (2018). Untargeted metabolomics approach (UPLC-Q-TOF-MS) explores the biomarkers of serum and urine in overweight/obese young men. Asia Pac. J. Clin. Nutr..

[31] Peterson J (2010). Dietary lignans: Physiology and potential for cardiovascular disease risk reduction. Nutr. Rev..

[32] Rodrigues FA (2014). Gingerol fraction from Zingiber officinale protects against gentamicin-induced nephrotoxicity. Antimicrob. Agents Chemother..

[33] González-Guardia L (2015). Effects of the Mediterranean diet supplemented with coenzyme q10 on metabolomic profiles in elderly men and women. J. Gerontol. A. Biol. Sci. Med. Sci..

[34] Sowers M (2008). Oestrogen metabolites in relation to isoprostanes as a measure of oxidative stress. Clin. Endocrinol..

[35] Wedekind R (2020). A metabolomic study of red and processed meat intake and acylcarnitine concentrations in human urine and blood. Am. J. Clin. Nutr..

[36] Holland WL (2007). Lipid mediators of insulin resistance. Nutr. Rev..

[37] Sampey BP (2012). Metabolomic profiling reveals mitochondrial-derived lipid biomarkers that drive obesity-associated inflammation. PLoS one.

[38] Cho K (2017). Combined untargeted and targeted metabolomic profiling reveals urinary biomarkers for discriminating obese from normal-weight adolescents. Pediatr. Obes..

[39] Liu N (2021). The role of oxidative stress in hyperuricemia and xanthine oxidoreductase (XOR) inhibitors. Oxid. Med. Cell Longev..

[40] Shin JH (2019). Differential effects of typical Korean versus American-style diets on gut microbial composition and metabolic profile in healthy overweight Koreans: A randomized crossover trial. Nutrients.

[41] Schulz KF, Altman DG, Moher D (2010). CONSORT 2010 statement: updated guidelines for reporting parallel group randomised trials. J. Pharmacol. Pharmacother..

[42] Schroeder N (2015). A randomized trial on the effects of 2010 Dietary Guidelines for Americans and Korean diet patterns on cardiovascular risk factors in overweight and obese adults. J. Acad. Nutr. Diet.

[43] Kim SA, Joung H, Shin S (2019). Dietary pattern, dietary total antioxidant capacity, and dyslipidemia in Korean adults. Nutr. J..

[44] The Korean Nutrition Society. Dietary Reference Intakes for Koreans. *Seoul: Korean Nutr. Soc.* (2010).

[45] Paik HY (2008). Dietary goals and dietary guidelines for Korean Adults. Korean J. Nutr..

[46] US Department of Agriculture. Sample Menus for a 2000 Calorie Food Pattern. Available online: https://naldc.nal.usda.gov/download/1333607/PDF Accessed on 24 May 2021.

[47] US Department of Agriculture. What We Eat in America.” Available online: https://www.ars.usda.gov/northeast-area/Beltsville-md-bhnrc/Beltsville-human-nutrition-research-center/food-surveys-research-group/docs/wweianhanes-overview Accessed on 24 May 2021.

[48] Jun S, Shin S, Joung H (2016). Estimation of dietary flavonoid intake and major food sources of Korean adults. Br. J. Nutr..

[49] Kim SA, Jun S, Joung H (2016). Estimated dietary intake of vitamin A in Korean adults: Based on the Korea National Health and Nutrition Examination Survey 2007–2012. J. Nutr. Health.

[50] Ahn S, Jun S, Kim SA, Ha K, Joung H (2017). Current status and trends in estimated intakes and major food groups of vitamin E among Korean adults: Using the 1–6th Korea National Health and Nutrition Examination Survey. J. Nutr. Health.

[51] Jun S, Chun OK, Joung H (2018). Estimation of dietary total antioxidant capacity of Korean adults. Eur. J. Nutr..

[52] Lee S, Lee DE, Singh D, Lee CH (2018). Metabolomics reveal optimal grain preprocessing (milling) toward rice koji fermentation. J. Agric. Food Chem..

