# Supplementary material for: Urine metabolomics unravel the effects of short-term dietary interventions on oxidative stress and inflammation: a randomized controlled crossover trial
Source: Sci Rep. 2024 Jul 3;14:15277. doi: 10.1038/s41598-024-65742-6 (PMC11222465; doi:10.1038/s41598-024-65742-6)
Supplement: Supplementary file 2 — Supplementary Information 2. [file 41598_2024_65742_MOESM2_ESM.docx]

**Urine metabolomics unravel the effects of short-term dietary interventions on oxidative stress & inflammation: a randomized controlled crossover trial**

**Digar Singh ^a, e, *^, Dongwoo Ham ^b, *^, Seong-Ah Kim ^c^, Damini Kothari ^d^, Yu Jin Park ^e^, Hyojee Joung ^b, f, †^, Choong Hwan Lee ^e, †^**

^a^ Department of Botany and Microbiology, Hemvati Nandan Bahuguna Garhwal University,

Srinagar (Garhwal), Uttarakhand 246174, India.

^b^ Institute of Health and Environment, Seoul National University, Seoul 08826, Republic of Korea.

^c^ Division of Economy and Society, The Seoul Institute, Seoul 06756, Republic of Korea.

^d^ Department of Biochemistry, Hemvati Nandan Bahuguna Garhwal University,

Srinagar (Garhwal), Uttarakhand 246174, India.

^e^ Department of Bioscience and Biotechnology, Konkuk University, Seoul 05029, Republic of Korea

^f^ Department of Public Health, Graduate School of Public Health, Seoul National University, Seoul 08826, Republic of Korea.

**^†^** Correspondence: HJ (e-mail: [hjjoung@snu.ac.kr](mailto:hjjoung@snu.ac.kr)) and CHL ([chlee123@konkuk.ac.kr](mailto:chlee123@konkuk.ac.kr))

**^*^** These authors contributed equally to this work.

**Funding**

This work was supported by the Research Program for Agricultural Science and Technology Development, National Academy of Agricultural Science, Rural Development Administration, Republic of Korea (Project no. PJ013475022019) and the National Research Foundation of Korea (NRF) grant funded by the Korea government (MSIT) (NRF-2023R1A2C1004930).

**Supplementary information**

Supplementary Figures S.1, S.2, & S.3, Supplementary Table S.1, and supplementary data 1 are provided with the manuscript and can be accessed using the “Supplementary data” link in the online posting of the article.

**Conflict of Interest**

All authors report no conflict of interest.

**Supplementary figures**


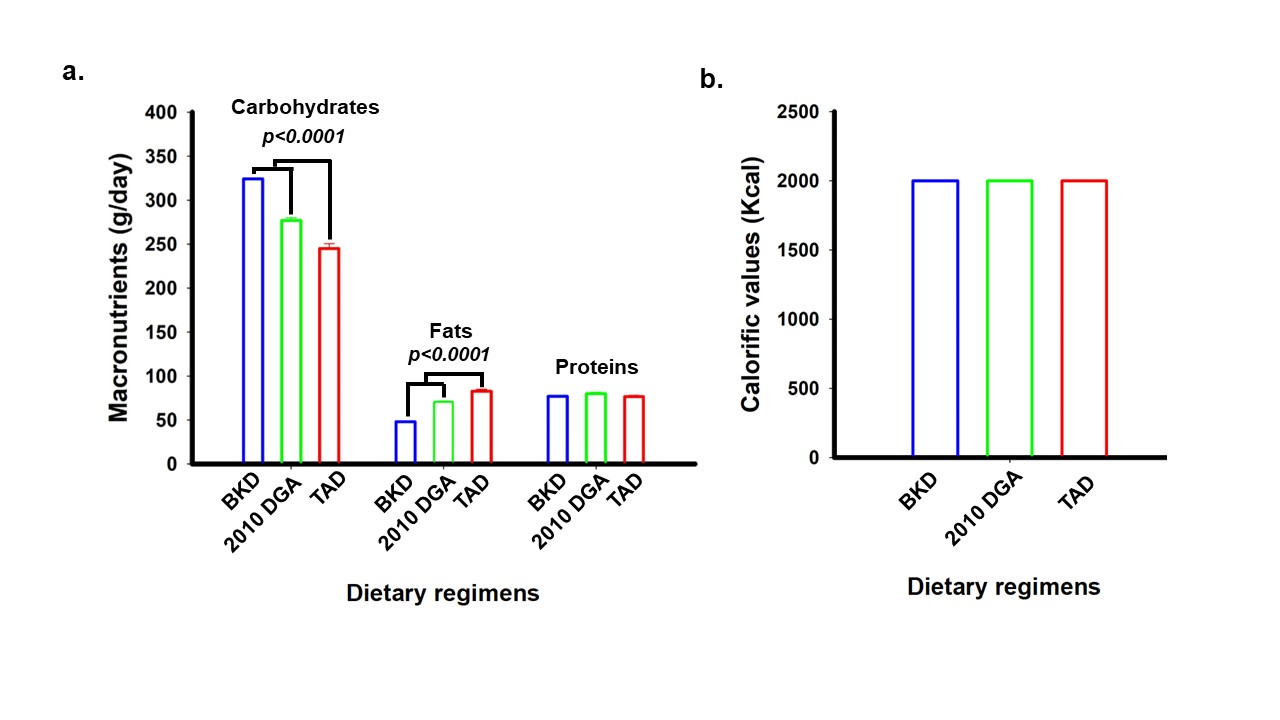


**Figure S.1.** Bar graph indicating the **(a)** macronutrient contents, and **(b)** calorific values for the three dietary regimens (BKD – Balanced Korean diet; 2010 DGA – Dietary guidelines for Americans, and TAD – Typical American diet) used in the study. The statistical significance for the data were expressed based on the Tukey’s post-hoc test. These components were computed based on the corresponding theoretical values estimated for the food components in each dietary regimen provide four times a day per week.


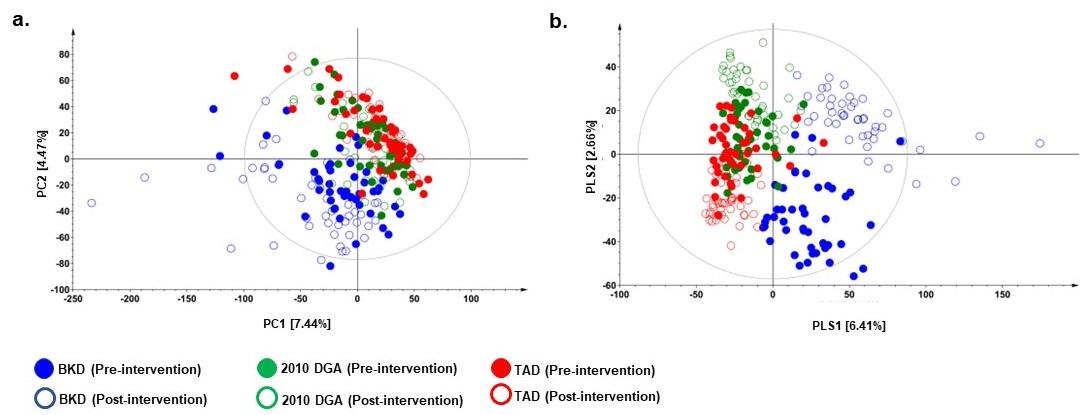


**Figure S.2.** The (a) PCA and (b) PLS-DA score plots based on the positive ESI mode UHPLC-LTQ-Orbitrap-MS/MS datasets representing metabolite profiles for the pre- (filled circles) and post-intervention (empty circles) urine samples collected from the participants enrolled under three different dietary regimens. The validation of this PLS-DA model's fit was confirmed with an R2X of 0.29 for model fitness regarding predictors, an R2Y of 0.95 indicating strong model fitness for response variables, and a respectable predictive performance, with Q2 at 0.67. The urine sample datasets representing the participant enrolled in three different dietary regimens is indicated with color codes, Blue color: BKD (Balanced Korean diet), Green color: 2010 DGA (2010 Dietary Guidelines for Americans), and Red color: TAD (typical American diet).


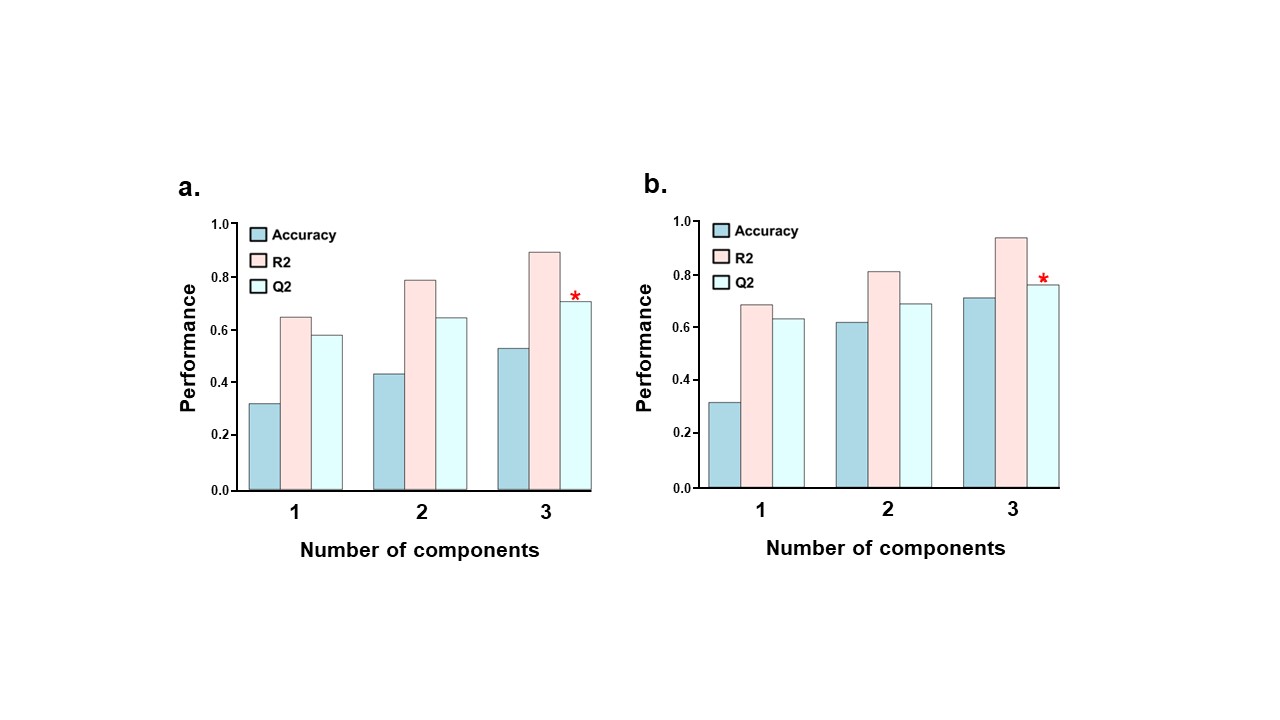


**Figure S.3.** The bar graph representing the leave-one-out- cross validation (LOOCV) analysis for the PLS-DA models based on the **(a)** negative, and **(b)** positive ESI mode UHPLC-LTQ-Orbitrap-MS/MS datasets representing the metabolite profiles for the pre- and post-intervention urine samples collected from the participants enrolled in dietary trials. Here, Q2 is an estimate of the predictive ability of the model, R2 (R-squared) explains the proportion of variance in the dependable variable predictable from the independent variable. Asterisk (*) represent that the selected model predicts reliably & significantly with 3 components. The analysis was performed on MetaboAnalyst 5.0 using the same datasets used in multivariate analysis.

**Supplementary table**

**Table S.1.** Estimated daily mean contents of antioxidants per 2,000-kcal serving portions from the three dietary regimens used in the study.

| **Micronutrient contents** | **BKD** | **2010 DGA** | **TAD** |
| --- | --- | --- | --- |
| Vitamin A (μg RAE) | 450.0 ± 61.0 | 627.6 ± 63.8 | 622.5 ± 86.5 |
| Retinol (μg) | 50.4 ± 4.8 | 225.8 ± 26.72 | 354.9 ± 37.9 |
| α-Carotene (μg) | 1,046.9 ± 394.2 | 544.6 ± 180.1 | 262.8 ± 122.4 |
| β-Carotene (μg) | 4,059.1 ± 565.7 | 4,418.4 ± 788.7 | 2,936.5 ± 918.8 |
| β-Cryptoxanthin (μg) | 425.9 ± 99.1 | 262.6 ± 89.7 | 289.6 ± 73.7 |
| Lutein/Zeaxanthin (μg) | 4,875.2 ± 1255.2 | 3,204.4 ± 1211.2 | 1,069.2 ± 138.8 |
| Lycopene (μg) | 13.5 ± 13.2 | 3,778.0 ± 966.6 | 6,527.6 ± 1665.6 |
| Vitamin C (mg) | 142.1 ± 15.4 | 195.4 ± 18.3 | 130.8 ± 20.0 |
| Vitamin E (mg α-TE) | 8.0 ± 0.4 | 12.2 ± 0.3 **** | 11.4 ± 1.1 |
| α-Tocopherol (mg) | 6.7 ± 0.4 | 11.2 ± 0.4 | 10.4 ± 1.0 |
| β-Tocopherol (mg) | 0.6 ± 0.08 | 1.1 ± 0.1 | 0.7 ± 0.1 |
| γ-Tocopherol (mg) | 8.5 ± 0.6 | 3.7 ± 1.1 | 5.3 ± 1.1 |
| δ-Tocopherol (mg) | 3.9 ± 0.7 | 3.9 ± 0.4 | 3.8 ± 0.8 |
| Flavonols (mg) | 32.7 ± 10.5 | 27.1 ± 2.1 ** | 15.2 ± 2.2 |
| Isoflavones (mg) | 22.8 ± 5.1 * | 4.6 ± 2.7 | 0.6 ± 0.1 |
| Flavones (mg) | 1.1 ± 0.4 | 3.1 ± 0.8 | 1.8 ± 0.6 |
| Flavanones (mg) | 8.2 ± 3.7 | 27.9 ± 9.8 | 25.4 ± 6.8 |
| Flavan-3-ols (mg) | 12.2 ± 2.6 | 20.3 ± 1.8 ** | 4.8 ± 2.4 |
| Anthocyanidins (mg) | 77.3 ± 24.7 | 134.3 ± 49.5 | 32.7 ± 31.3 |
| Proanthocyanidins (mg) | 137.5 ± 35.5 | 358.4 ± 65.2 ** | 71.8 ± 23.5 |

^†^ Abbreviations: BKD, balanced Korean diet; 2010DGA, 2010 Dietary Guidelines for Americans; TAD, typical American diet; RAE, retinol activity equivalents; TE, tocopherol equivalents. The statistical significance for the data were expressed based on the two-way ANOVA and Tukey’s post-hoc multiple comparison test (**** *p*<0.0001, ** *p*<0.01, * *p*<0.05).
